# Video Didactic Preparation Augments Problem-Based Learning for First Year Medical Students

**DOI:** 10.1177/23821205231177862

**Published:** 2023-05-29

**Authors:** Kelly L Hamilton, Yu-Chun Kuo, Peter Horneffer, T Peter Stein, Gary S Goldberg

**Affiliations:** 1 Departments of Molecular Biology and Surgery, Rowan University Graduate School of Biomedical Sciences and School of Osteopathic Medicine, Stratford, NJ, USA; 2 71541 Psychiatry Program, Medstar Georgetown University Hospital, Washington, DC, USA; 3 Department of Science, Technology, Engineering, Art and Mathematics Education, 3536 Rowan University, Glassboro, NJ, USA; 4 268547 Dean's Office, All American Institute of Medical Sciences, Black River, St Elizabeth, Jamaica

**Keywords:** Case-based learning, PBL, problem-based learning, readiness, student perception, virtual patient cases

## Abstract

Problem-based learning (PBL) utilizes a self-directed strategy. This process relies on group participation to succeed. Students without a background in biology or medicine can feel overwhelmed by the complexity of the subject matter and unable to participate in the group learning process. We incorporated curated educational videos in the PBL curriculum to help address this situation. First year medical students participated in this study in the form of a typical PBL session. They were then assessed on basic and clinical science knowledge and their learning experience. Student basic science and clinical knowledge were similar between the student groups. However, the students given a list of suggested videos scored higher in their learning experience, perception of feeling prepared, and participating in the group PBL experience than students who were not given the video list. Results from this study indicate that videos can be utilized to enhance the PBL process.

## Introduction

Problem-based learning (PBL) employs a self-directed strategy. The process is designed to motivate students to explore and understand concepts integral to their curriculum. In contrast to conventional classes led by a didactic process, PBL utilizes a flipped classroom where students learn topics to address problems related to their field of study. PBL offers significant advantages over didactic lectures as students learn material to address real life situations. However, the process also presents significant challenges with respect to student readiness to learn.^1–3^

PBL classes typically consist of small groups of 6 to 12 students who investigate and discuss topics with each other. In the context of undergraduate medical education (UME), PBL uses clinical cases that students work their way through and craft their own learning objectives. This process motivates the learner to use a combination of critical thinking, content searching, and application of information to clinical situations.^1–3^

UME has been consistently evolving since the rise of internet based educational resources including online lectures and question banks. In addition, the spread of COVID-19 has influenced education in many ways. In particular, the pandemic has precipitated significant changes in course delivery methods from in person to online learning modalities in medical education and clinical training.^4,5^ Basically, the COVID-19 pandemic prompted medical schools to implement more online learning components. This has led to an expansion of online resources that students supplement with their school curriculum. Results from several studies have documented the effectiveness of online learning modalities in medical education.^5^ These innovations benefit many students, particularly those with backgrounds in medical or biological science. However, students without experience in biological foundations can struggle with connections between the curriculum content and clinical applications presented in class. These students can feel unprepared and less able to participate in the PBL process than their more experienced peers.^2,6^

Overall results from research support the use of PBL as a pedagogical strategy to facilitate student learning in online settings.^7,8^ Applying PBL in online learning environments can enhance student interaction, collaboration, discussion, and participation in class, as well as student performance, critical thinking, and problem-solving skills.^8–10^ Results from some studies indicate that technology supported PBL, or the use of PBL strategies in online settings, has a positive impact on student performance.^9,10^ For example, Saqr et al investigated online PBL courses in dental education and found that interaction variables of the PBL process significantly predict student performance.^10^ In addition, Aslan conducted a study to examine the use of PBL and teacher based methods for 45 students in online classes to find that students in the PBL group performed significantly better in learning achievement tests than those in the group with a traditional teacher based method.^9^

Evidence also indicates that students in medical education or related fields have positive attitudes towards the use of PBL in online settings.^11,12^ Ezra et al investigated preclinical dental medicine student perceptions of the effectiveness of PBL using online learning methods. More than 50% of the students reported that PBL is an effective method for online courses.^11^ In addition, Gould and Sadera found that students in health care professional training reported a positive learning experiences with online PBL modules, as well as the potential of PBL in increasing their ability to apply their learning to clinical practice.^12^ Moreover, results from studies indicate that online PBL can promote content knowledge as effectively as face-to-face PBL session.^12,13^

Perceived preparedness has been associated with higher student performance. In addition, utilization of a defined and finite set of learning resources also correlates with higher student performance.^6^ It can be difficult to evaluate learning resources in a PBL curriculum. The PBL process is self driven, and a student can find themselves wandering through a wide array of online resources and videos without benefit.^2,6^ We sought to provide guidance to these students in order to increase their learning efficiency. We constructed a set of curated lecture videos designed to present content related to the clinical material they were presented with. These videos were offered as an elective list to maintain the independent learning structure of the PBL approach.

This study was performed to determine if front-loading concise basic science and clinical information using faculty selected videos mapped to interactive virtual patient cases in a PBL environment leads to improved classroom preparedness and satisfaction. We hypothesized that PBL students, especially those without a background in biological or medical sciences, would benefit from some degree of didactic support such as that provided by focused media in the form of this curated set of videos.

## Methods

### Period of study and participant recruitment

This study was performed between September of 2021 and July of 2023. Thirteen first year medical students participated in this study. These volunteers were recruited by written informed consent with IRB approval from the Rowan University School of Osteopathic Medicine (PRO-2021-507 approved September 3, 2021). All students were in good academic standing and were recruited without bias or compensation to represent a typical group and study session. Students were placed into experimental (*n* = 5) or control (*n* = 8) groups by 1:1 randomization. These students were blinded such that they did not know what other students would be participating in the study, or what group the other participants were in throughout the study. Study subjects participated in a typical 2.5 hours in person PBL session run as a component of the Rowan-SOM curriculum at the Stratford NJ campus. Both (control and experimental) groups were managed together in the same session to control for variables including, time, and day.

### Patient case content

A patient case on the PBLMed (pblmed.com) online platform was utilized for this study. Virtual patients on this platform are presented with personalized answers to over 220 questions (including description of present condition along with medical, social, and family history), 130 exams (including cardio, pulmonary, skin, reflexes, palpation, strength, motion, and sensory findings), and 640 tests (including imaging and lab values with clear reports and summaries). Students treated this virtual patient to formulate diagnosis and treatment plans based on this information. The cases are complex and include multiple encounters which are summarized in a case “snapshot” that is seen at the end of the session. The case generated a wide array of learning objectives in basic and clinical science (including cell and molecular biology, physiology, pharmacology, imaging, social factors, and ethics).

### Supplemental video selection

Media provided by the Lecturio (lecturio.com) online platform was utilized for this study. The Lecturio platform presents a comprehensive library with over 7500 videos covering the medical school educational curriculum. These videos are supported by over 20 000 questions designed to enhance the learning process relevant to core principles of the subject matter. The Lecturio platform combines learning technology with comprehensive monitoring and assessment features in this manner. Each video is approximately 5 to 10 minutes long, and followed by a few focused questions designed to aid in learning and retention.

A list of videos was mapped by keywords related to basic science including biochemistry, genetics, and metabolism, as well as clinical science including symptoms, diagnosis, and treatment plans involving the patient case. Students in the experimental group were provided with this list of content specific videos 72 hours before the PBL session. This list was not provided to students in the control group. While the media focused on topics related to virtual case, they did not reveal content or diagnosis of the case. Students were free to choose which videos they watched. In this way, they were motivated to prepare for the session by self driven independent study in this spirit of the PBL method.

### Assessment and data analysis

An assessment exam was administered to students after they finished the patient case presentation. The exam consisted of 30 questions. These included 14 questions to assess understanding of basic science, 10 questions to assess understanding of clinical knowledge related to case, and 6 experiential questions to assess student perception of readiness and the PBL learning experience. These experiential questions asked if students: (a) felt prepared to participate in the case that was presented, (b) learned “a lot of material” during the PBL session, (c) found that presentation and participating in group increased their understanding of the subject material, (d) felt that presentation and participating in group learning of the case motivated them to learn more about the subject material, (e) felt that presentation and participating in group learning of the case was a good and productive use of their time as a medical student, and (f) want their school to use a PBL learning platform as part of our curriculum. Results were analyzed to evaluate differences between the experimental and control groups by *t*-test with Graphpad Prism (version 9.4).

This study was designed to assess the ability of curated set of videos to enhance the PBL patient case-based learning process. A list of 60 videos relevant to the case was provided to students in the experimental group. These videos were focused on topics including genetics (10 videos), clinical presentation (32 videos), metabolism (3 videos), pharmacology (6 videos), and diagnosis (9 videos). These students were able to choose which of these videos they wanted to watch during a 72-hour time period prior to the case presentation during a typical PBL session.

## Results

Thirteen students participated in this study. Eight of these students were in the control group and did not watch assigned videos. Five students were in the experimental group and watched 4 to 26 assigned videos amounting to 36 to 176 minutes before the PBL session. These students in the experimental group watched an average of 10.8 ± 3.9 videos over an average of 78 ± 25 minutes (mean ± SEM, *n* = 5).

**Figure 1.**
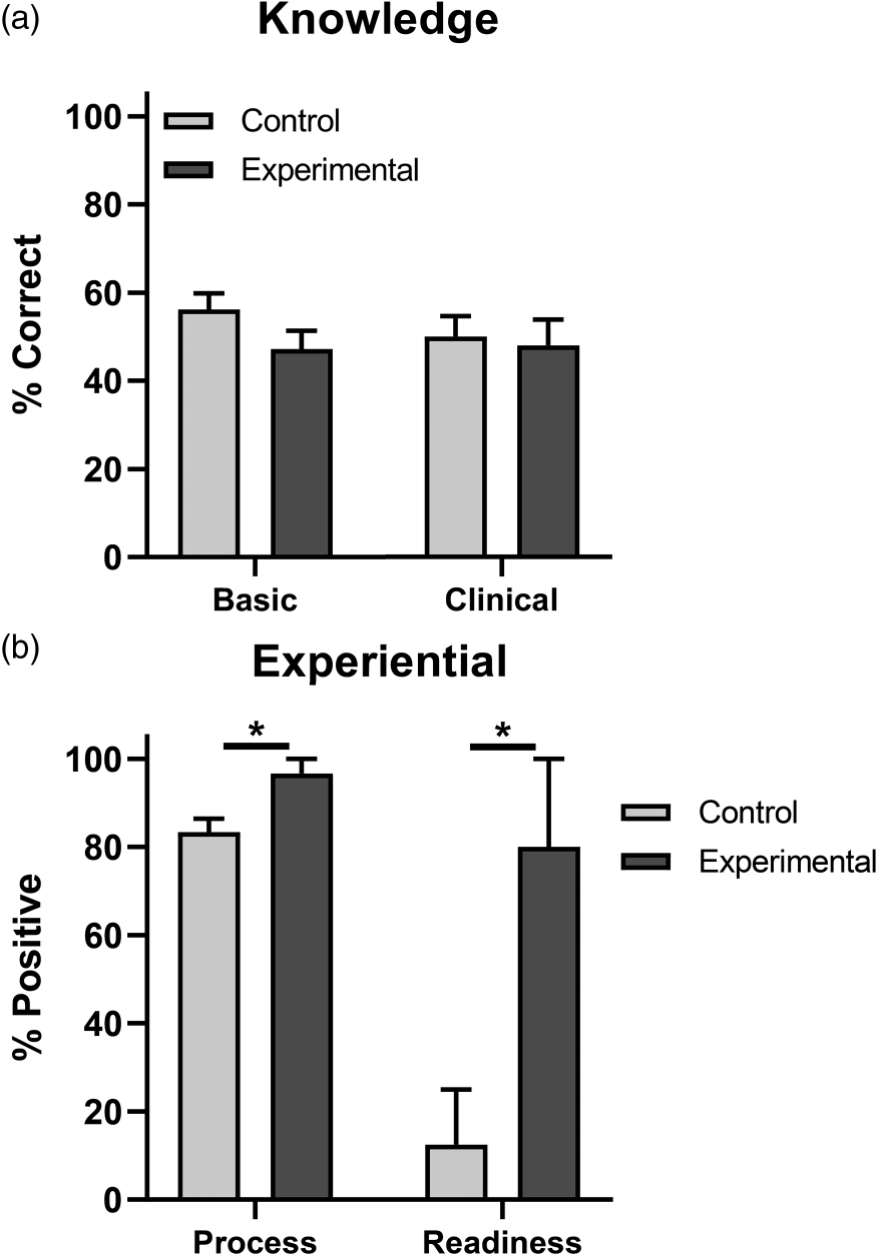
Effect of front loaded videos prior to a case presentation on student basic and clinical science knowledge, and learning experience and perception of readiness, during a single PBL session. Data are shown at percent of knowledge based questions answered correctly in panel a, and experiential questions answered positively (affirmative) in panel b, by students in the experimental group which were provided with a list of curated videos pertinent to the case, and the control group which were not provided with this list (mean ± SEM) as indicated. Asterisks indicate *p* < 0.05 by *t*-test.

Students were given an assessment exam after finishing the case presentation in the PBL session. Results from this study found no significant differences between students in the control and experimental group on performance on basic science or clinical questions as shown in Figure 1a. Students in the control group scored 56 ± 3.7% and 50 ± 4.6% (mean ± SEM, *n* = 8), while students in the experimental group scored 47 ± 4.2% and 48 ± 5.8% (mean ± SEM, *n* = 5) on basic science and clinical questions, respectively. Values from these groups were found to be insignificant from each other (*p* > 0.05 by *t*-test).

Although students in control and experimental groups performed similarly on basic science and clinical knowledge questions, students in the control group performed better on experiential questions as shown in Figure 1b. Students in the control group scored 83 ± 3.1% (mean ± SEM, *n* = 8), while students in the experimental group scored 97 ± 3.4% (mean ± SEM, *n* = 5) on these questions. Therefore, students who watched assigned videos performed 13% better than students that did not watch these videos on experiential questions (*p* < 0.05 by *t*-test).

The effect of supplemental videos on PBL session experience was most evident in the student perception of class preparedness as shown in Figure 1b. Only 1 of the 8 students (12.5%), in the control group felt prepared for the case presentation in class, while 4 of the 5 students in the experimental group felt prepared (*p* < 0.05 by *t*-test). This was apparently the result of watching the supplemental videos since students in the experimental group did not perform better on basic science or clinical questions than those in the control group.

Interestingly, 100% of the students in this study reported that they wanted a virtual PBL medical program incorporated into their learning curriculum. This was seen in both the control and experimental groups. These data indicate that students appreciated the patient case presentation regardless of their background knowledge or perception of readiness.

## Discussion

PBL in medical education utilizes a self-directed strategy to study biological and clinical sciences. Students typically work in small groups to solve a virtual patient case. Cases are chosen to present specific basic and clinical science concepts that generate a core class curriculum.

Group participation is a needed for the PBL process to succeed. Students with a background in medicine or biology tend to enhance the group learning dynamic. However, students without this experience can become overwhelmed by the complexity of the patient cases and subject matter. These students feel less able than their more experienced peers to participate in the PBL learning process. We incorporated a focused list of curated educational videos that could be accessed by internet in the PBL curriculum to help address this situation. The nature and details of patient cases were not revealed, and students were free to decide if and when to watch these videos in order to maintain an independent learning strategy essential to the PBL process.

First year medical students participated in this study in the form of a typical PBL session. They were then assessed on basic and clinical science knowledge and their learning experience. Student basic science and clinical knowledge were similar between the experimental and control groups. However, the experimental group scored 13% higher in their learning experience, and 400% higher in their perception of feeling prepared and participating in the group PBL experience.

This initial study presents some limitations. It is a single-center study with a limited sample size, questionnaire pilot validation, and sample size power analysis was not performed. In addition, participants were not blinded to whether they were in the control or experimental group, which might have influenced their answers to experiential questions. Nonetheless, results from this study indicate that a limited set of curated videos can be mapped to virtual patient cases in order to enhance the PBL process. Taken together, these data indicate that front-loading media can augment student perception of their preparedness to participate and learn from PBL case presentations.
